# A review of unusual species of *Cotesia* (Hymenoptera, Braconidae, Microgastrinae) with the first tergite narrowing at midlength

**DOI:** 10.3897/zookeys.580.8090

**Published:** 2016-04-12

**Authors:** Ankita Gupta, Mark Shaw, Sophie Cardinal, Jose Fernandez-Triana

**Affiliations:** 1 ICAR-National Bureau of Agricultural Insect Resources, P. B. No. 2491, H. A. Farm Post, Bellary Road, Hebbal, Bangalore,560 024, India; 2 National Museums of Scotland, Edinburgh, United Kingdom; 3 Canadian National Collection of Insects, Ottawa, Canada

**Keywords:** Cotesia trabalae, new species, Trabala vishnou, India, Cotesia pistrinariae, Mylothris chloris, Africa

## Abstract

The unusual species of *Cotesia* (Hymenoptera, Braconidae, Microgastrinae) with the first tergite narrowing at midlength are reviewed. One new species, *Cotesia trabalae* **sp. n.** is described from India and compared with *Cotesia pistrinariae* (Wilkinson) from Africa, the only other species sharing the same character of all the described species worldwide. The generic placement of these two species, based on molecular and morphological analyses as well as parasitoid biology is discussed.

## Introduction

With 269 described species (Fernandez-Triana and Ward 2015), *Cotesia* is the second largest genus in the hyperdiverse subfamily Microgastrinae (Hymenoptera, Braconidae), with estimates of its actual diversity ranging from 1,500 (Mason 1981) to 2,500 species (van Achterberg and Polaszek 1996). The genus was described as monotypic by Cameron in 1891, but soon after it was synonymized under *Apanteles* (Szépligeti 1904: 105) and remained that way until Mason (1981) reinstated it as valid and transferred a number of species to it. Mason estimated that 30-40% of the temperate species previously considered as ‘*Apanteles*’ actually belong to *Cotesia*, while in tropical areas that proportion is only 10-20% (Mason 1981: 113). Austin and Dangerfield (1992: 21) considered *Cotesia* to be the largest genus within Microgastrinae – although that assumption is questionable, based on described and undescribed species available in collections it is clear that *Apanteles* and *Glyptapanteles* are much more diverse, especially in the tropics. Regardless, *Cotesia* comprises a huge assemblage of species and it is found in all biogeographical regions of the planet (Yu et al. 2012).

In spite of its diversity, species of *Cotesia* tend to be relatively uniform morphologically, especially regarding the shape of tergites 1–3 and propodeum sculpture. When redescribing the genus, Mason (1981: 110–111) stated ‘…Tergite I occasionally wider than long but usually a little longer than wide and broadened apically, occasionally somewhat barrel-shaped or parallel-sided, but never narrowed apically; never with a median apical depression… Tergite I frequently smooth basally but the posterior part almost invariably rugose or rugopunctate… Propodeum invariably rugose and never with an areolet; usually with a median longitudinal carina that may be partially obscured by rugosity and usually an incomplete transverse carina laterally separating the rugose declivity from a smoother anterior area…’

Until now only one species of *Cotesia* was known to have a significantly different shape of mediotergite 1 (henceforward abbreviated as T1). The species *Cotesia pistrinariae* (Wilkinson, 1929) has T1 strongly narrowing at midlength so that T1 width medially (at narrowest point) is 0.5–0.6× its width at anterior margin and 0.6–0.7× its width at posterior margin. The shape of T1 was so bizarre that in the original description of the species, as ‘*Apanteles pistrinariae*’, Wilkinson (1929: 445) wrote ‘…The unusual form of the 1st tergite, although a character whereby the species may be immediately separated from all others, renders the satisfactory placing of this species in my key a difficult matter…’ Even at that time, when species of *Cotesia* were still considered to be part of a much expanded ‘*Apanteles*’ genus, this species was hard to place within a group.

In our studies of the world fauna of Microgastrinae we have found a new species of *Cotesia* with similar shape of T1 (narrowing at midlength), which is described below, together with diagnostic characters to separate it from *Cotesia pistrinariae*. We discuss further the generic placement of those two species, based on molecular and morphological analyses as well as parasitoid biology.

## Methods

This paper is based on study of *Cotesia* specimens collected in India (housed at the ICAR-National Bureau of Agricultural Insect Resources (NBAIR), Bangalore, India); and Africa (housed in the National Museums of Scotland (NMS), Edinburgh, United Kingdom).

Morphological terms and measurements of structures are mostly as in Mason (1981), Huber and Sharkey (1993), and Whitfield (1997).

Photos of the Indian species were taken with a Leica M 205 A stereozoom microscope with Leica DC 420 inbuilt camera using automontage software (version 3.8). Photos of the African species were taken with a Keyence VHX-1000 Digital Microscope, using a lens with a range of 13–130 ×. Multiple images through the focal plane were taken of a structure and these were combined to produce a single in-focus image using software associated with the Keyence System.

DNA barcodes were obtained using DNA extracts prepared from single legs using a glass fibre protocol (Ivanova et al. 2006). Briefly, total genomic DNA was re-suspended in 30 μl of dH2O, and the standard barcoding region near the 5’ terminus of the COI gene was amplified using standard primers (LepF1– LepR1) following established protocols (Smith et al. 2006, 2007, 2008). If the initial amplification was unsuccessful, shorter sequences were generated using internal primers and subsequently contigued together. A MUSCLE sequence alignment was generated in Geneious 8.1.7 (http://www.geneious.com, Kearse et al. 2012) for 241 species of *Cotesia* with sequences over 600 base pairs available in the Barcode of Life Data System (BOLD, http://www.boldsystems.org/) (Ratnasingham and Hebert 2007). In addition, sequences from species of most other genera of Microgastrinae were used as outgroups when available (120 outgroup sequences). All information for individual specimens in BOLD can be retrieved by Process ID (sequence accession) or Sample ID (voucher codes); and the newly described species can be retrieved from Genbank (codes KJ459172, KJ459169, KM875666, KT308157 and KT308158) (information summarized in Table 1 for the new species).

**Table 1.** Showing comparative measurements from different localities.

Female characters	Kasaragod	Shimla	Meghalaya
Body length in mm	2.59, 2.51, 2.48, 2.44, 2.58, 2.60	2.62, 2.66, 2.43	2.73
Fore wing length	2.42 (for body length 2.59 mm) 2.23 (for body length 2.51 mm)	2.59 (for body length 2.62 mm)	2.70
Antenna length/body length	2.546 (for body length 2.59 mm) 2.438 (for body length 2.51 mm)	2.52 (for body length 2.62 mm)	
Ratio of ocular-ocellar line/posterior ocellus diameter	1.50	2.00−2.03	1.89
Ratio of interocellar distance/posterior ocellus diameter	1.83	1.98−2.02	2.23
Antennal flagellomere 2 (ratio of length/width)	3.50	2.36−3.05	2.73
Antennal flagellomere 14 (ratio of length/width)	1.83	1.91−1.92	1.92
Ratio of length of flagellomere 2/length of flagellomere 14	1.91	1.83−1.85	1.59
Ratio of metafemur length/width	3.47	3.57−4.20	3.30
Number of pits in scutoscutellar sulcus	9	9	9
Ratio of mediotergite 1 width at anterior margin/width at posterior margin:	1.01–1.05	0.77−0.78	0.88
Ratio of mediotergite 1 median width/ width at posterior margin	0.86–0.92	0.77−0.81	0.83
Ratio of mediotergite 2 width at posterior margin/length	2.87−2.94	2.26−2.32	2.32
Ratio of ovipositor sheaths length/metatibial length	0.16	0.21	0.16−0.17
Ratio of metatibia inner spur length/metabasitarsus length	0.66	0.57−0.59	0.61
Ratio of maximum height of mesoscutellum lunules/maximum height of lateral face of mesoscutellum	0.30	0.45	0.37
Ratio of length of fore wing veins r/2RS	1.22	0.75−0.77	1.06
Ratio of length of fore wing veins 2RS/2M:	1.44	1.64−1.78	1.73
Ratio of length of fore wing veins 2M/(RS+M)b	1.25	1.25−1.38	1.25
Pterostigma (ratio of length/width)	2.53	2.52−3.00	2.55
Ratio of lengths: meta basitarsus/inner metatibial spur/outer metatibial spur	0.35/0.23/0.15	0.37/0.21/0.14, 0.37/0.22/0.14	0.43/0.26/0.15

Both ends of the sequence alignment were trimmed to reduce missing data and a neighbor-joining tree based on Kimura 2-parameter distances was generated in Geneious 8.1.7. A Bayesian majority rule consensus tree was generated in MrBayes 3.2.1 (Ronquist et al. 2011). To find the best-fit partitioning scheme and models of molecular evolution for the nucleotide alignment, PartitionFinder v1.1.1 (Lanfear et al. 2012) was used. Two independent runs of 20 million generations in which each codon position formed a partition with a GTR+IG model applied (based on results of PartitionFinder analysis) were analysed. To ensure that both runs had converged and reached stationarity, trace files of all estimated parameters were observed and the estimated sample size of each parameter was verified to be over 200. The first 10% of samples were removed as burn-in.

## Results

**Figure 1.**
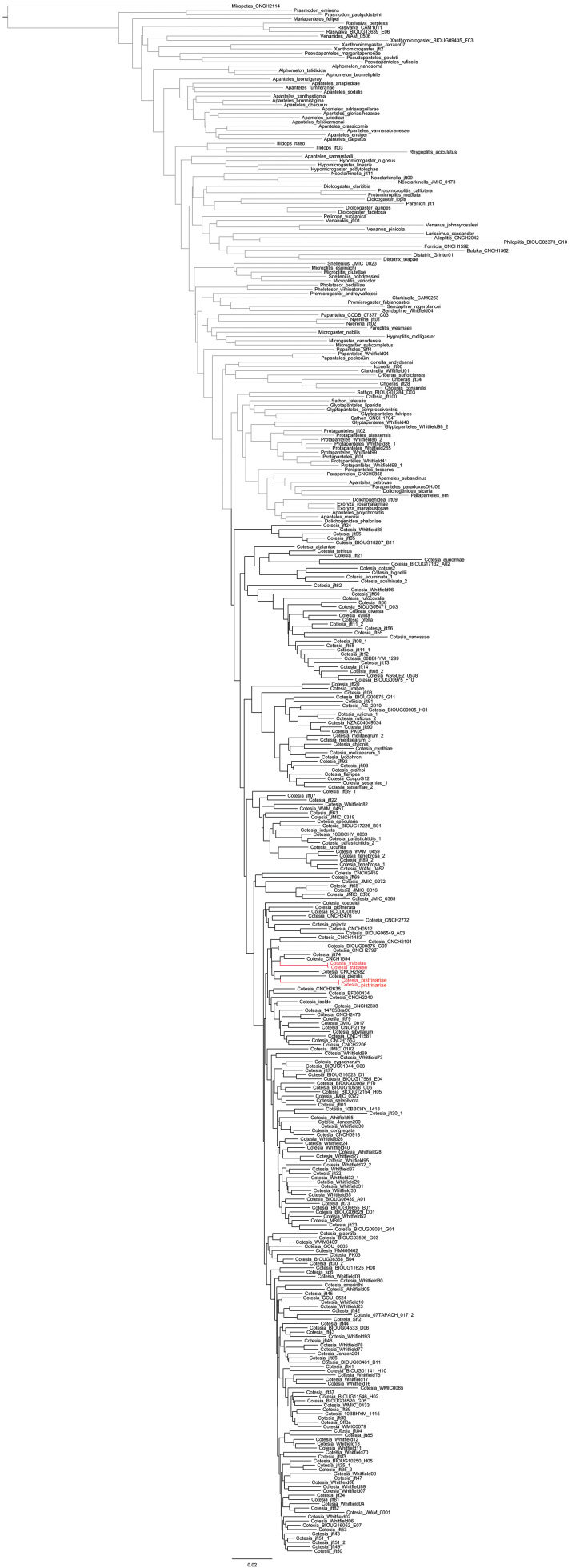
Neighbor-joining tree based on Kimura 2-parameter distances of 241 species of *Cotesia* and 120 species of other genera of Microgastrinae. The two *Cotesia* species known to have T1 narrowing at midlength are colored in red.

**Figure 2.**
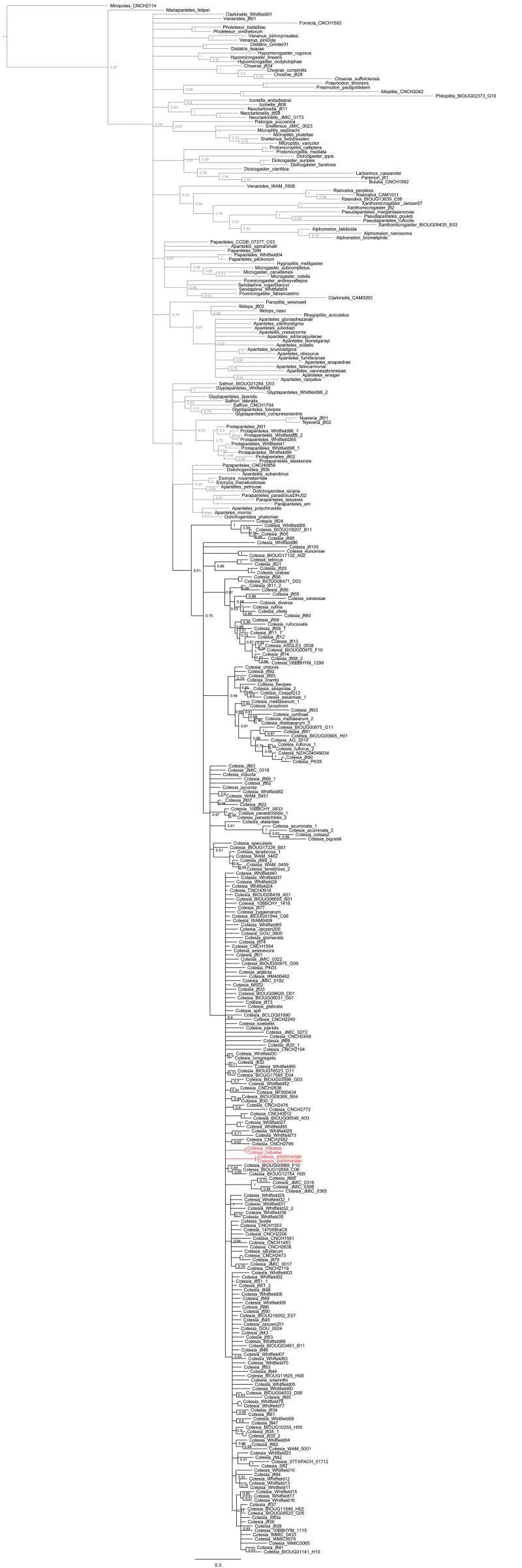
Bayesian majority rule concensus tree of 241 species of *Cotesia* and 120 species of other genera of Microgastrinae. The two *Cotesia* species known to have T1 narrowing at midlength are colored in red.

To date, only two species of *Cotesia* are known to have a T1 narrowing at midlength. That represents less than 1% of all described species worldwide. In the neighbor-joining tree both species cluster more closely with other species (Fig. 1), and in the Bayesian tree (Fig. 2) they are part of a large unresolved polytomy which provides no support for them being sister species, although it does not preclude that possibility either. However, the molecular data support the monophyly of *Cotesia*, including both *Cotesia pistrinariae* and *Cotesia trabalae*.

**Figure 3.**
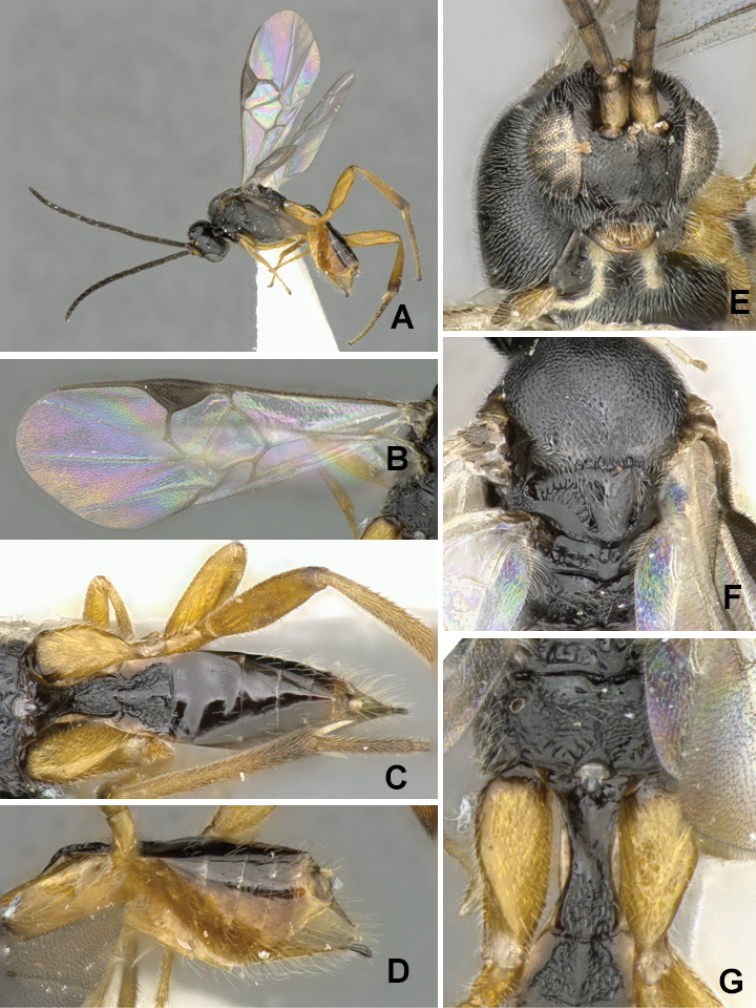
*Cotesia pistrinariae*. **A** Habitus, lateral view **B** Fore wing **C** Metasoma, dorsal view **D** Metasoma lateral view **E** Head, frontal view **F** Mesosoma, dorsal view **G** Details of propodeum, T1 and T2, dorsal view.

*Cotesia pistrinariae*, from Africa (Fig. 3), has the propodeum with transverse and median carinae weakly defined and only partially visible, and without traces of lateral carinae or areola. The hypopygium is relatively large (clearly protruding beyond apex of metasoma) and with numerous and long setae. T1 is narrower: at approximately half its length it is 0.5–0.6 × as wide as its width at the anterior margin and 0.6–0.7 × its width at the posterior margin of the tergite. The species is rather widely distributed in Africa (Cape Verde Islands, Democratic Republic of Congo, Eritrea, Ethiopia, Malawi, Nigeria, Rwanda, South Africa). We observed slight differences in coloration among specimens from different countries, but could not find any evidence to separate them and thus all are considered here to belong to the same species –although future studies might change that. All known caterpillar hosts belong to the family Pieridae (*Mylothris chloris* (Fabricius) and undetermined gregariously feeding species).

**Figure 4.**
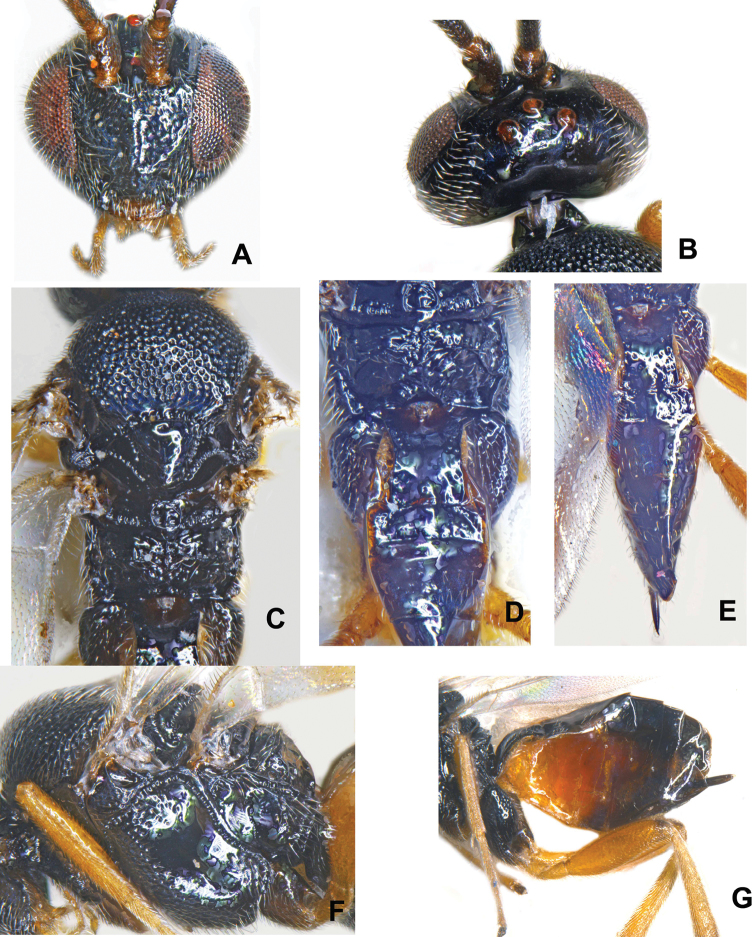
*Cotesia trabalae* sp. n. (Kasaragod): **A** Head in frontal view **B** Vertex **C** Mesosoma with propodeum in part **D** Propodeum with metasoma in part **E** Metasoma **F** Mesopleuron **G** Metasoma in lateral view.

**Figure 5.**
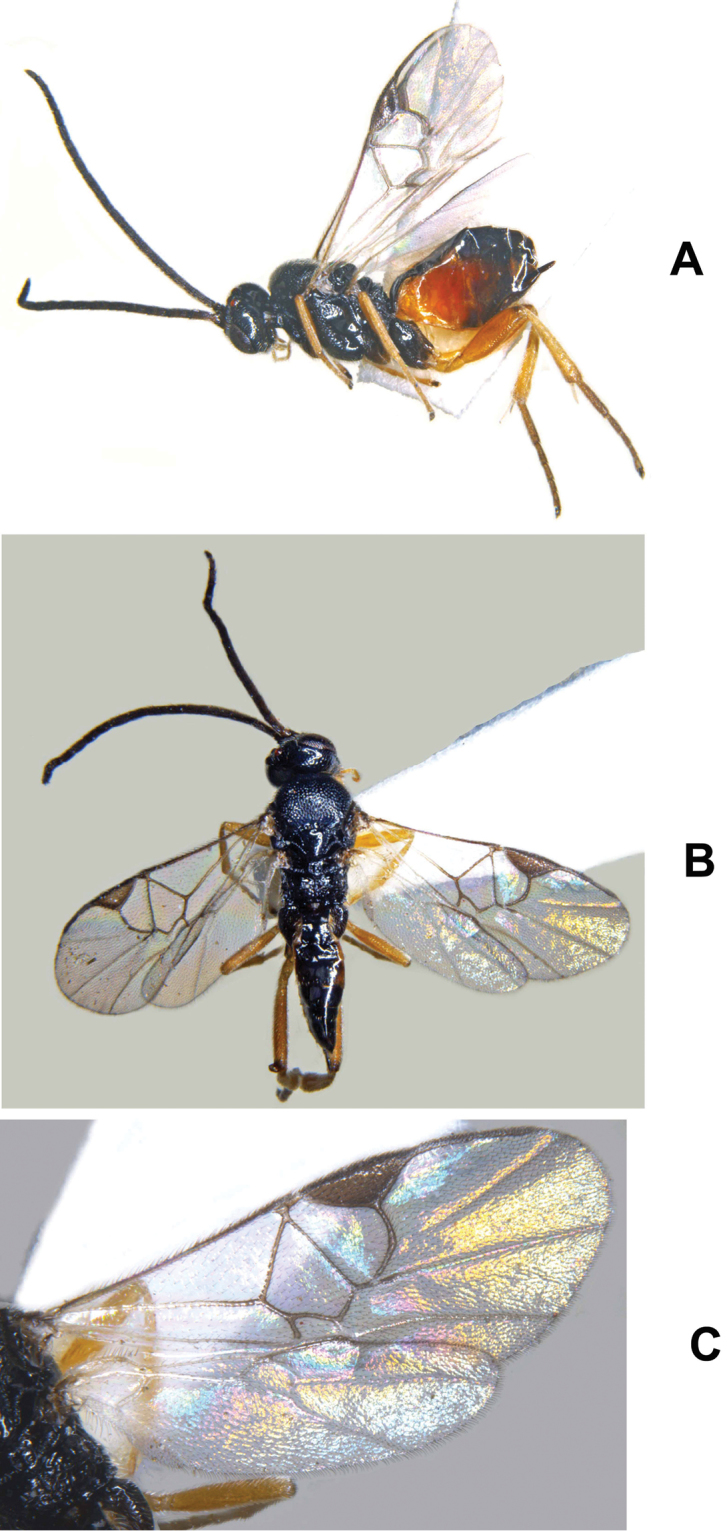
*Cotesia trabalae* sp. n. (Kasaragod): **A** Female in habitus **B** Female in dorsal view **C** Wings.

**Figure 6.**
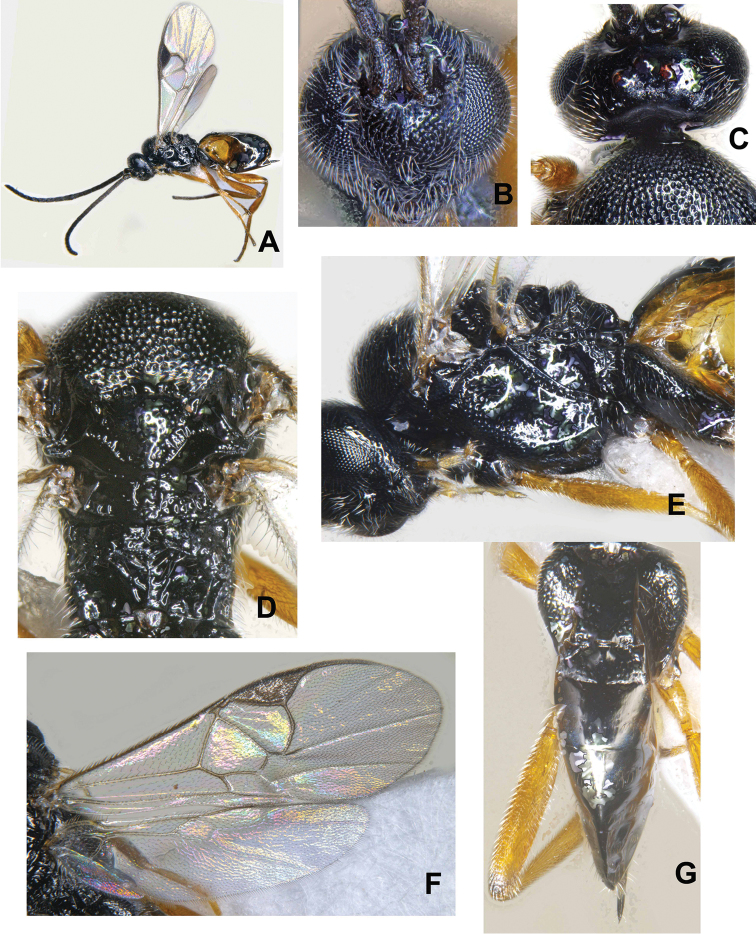
*Cotesia trabalae* sp. n. (Shimla): **A** Female in habitus **B** Head in frontal view **C** Vertex **D** Mesosoma with propodeum in part **E** Mesopleuron **F** Wings **G** Metasoma

The Indian species, *Cotesia trabalae* sp. n., described below, is obviously different (Figs 4–6). The propodeum has transverse and median carinae which are clearly defined and complete, as well as two partial lateral carinae on the posterior half of the propodeum (which seem to define a partial areola). The hypopygium is relatively small (not protruding beyond apex of metasoma) and mostly without setae. T1 is wider than in *Cotesia pistrinariae*; its narrowest width, at approximately the half length of the tergite, is 0.8 × (rarely up to 0.9 ×) its width at the anterior and posterior margins of the tergite. The species is known only from India. The caterpillar hosts belong to Lasiocampidae (*Trabala vishnou* (Lefèbvre)).

The carination pattern on the propodeum of *Cotesia trabalae* is rather unusual. According to Mason’s definition of the genus, *Cotesia* never has an areola on the propodeum (Mason 1981: 111), although this could be argued against, as certain species currently included in the genus seem to have a similar carination pattern to that found in *Cotesia trabalae* [see, for example, illustrations of the propodeum for the species *Cotesia rubripes* (Haliday, 1834) and *Cotesia lineola* (Curtis, 1830), as detailed by Wilkinson (1945, figs 27 and 57 in that paper)].

The definition and limits of the genus *Cotesia* are beyond the scope of this paper and will require a comprehensive study of the world fauna – including closely related genera such as *Protapanteles*. But for the time being we are considering all of the species dealt with in this paper as belonging to *Cotesia* based on the available evidence. In spite of the unique shape of T1 (and the rather unusual carination pattern of the propodeum in *Cotesia trabalae*), the rest of the morphological characters analyzed strongly suggest that those two species are best placed in *Cotesia*. The molecular data also support the monophyly of the genus (Figs 1, 2).

### Cotesia trabalae

Taxon classificationAnimaliaHymenopteraBraconidae

Gupta sp. n.

http://zoobank.org/6DEF35EA-D67F-4AF7-B50F-1ABF5A4FF28C

Figs 4 , 5 , 6

#### Type material.

Holotype ♀ (NBAIR), INDIA, Kerala, Kasaragod, 12.5013°N; 74.9900°E, 10.xii.2013, ex: caterpillar of *Trabala vishnou* (Lefèbvre), NBAIR, Code 101213, DNA Voucher−BR-2014 (NBAIR).

#### Specimens examined.

Paratypes: 5 ♀ (NBAIR) [part of the same brood as holotype]; 5 ♀ (NBAIR), INDIA: Himachal Pradesh, Shimla, 30.viii.2014, ex: caterpillar of *Trabala vishnou* (Lefèbvre) on *Rubus* sp.; 3 ♀ (NBAIR), INDIA: Meghalaya, Barapani, 25.x.2014, ex: caterpillar of *Trabala vishnou* (Lefèbvre) on *Ricinis communis* L.

#### Description.

**Female** (Figs 5A, 6A). Body in lateral view: not distinctly flattened dorso–ventrally. Body length (head to apex of metasoma): 2.43–2.66 mm to 2.73 mm. Fore wing: length: 2.42 (for body length 2.59 mm), 2.23 (for body length 2.51 mm).

*Color*. Body mostly black except for yellowish brown sternites in anterior half. Antenna color: scape, pedicel, and flagellum dark. Pro- and meso- coxae color: brown. Meta- coxa color: black. Pro- and meso- femur color: yellow. Meta- femur color: yellow, except for dark brown coloration on extreme apical tip. Metatibia and metatarsus color: yellowish brown. Tegula and humeral complex color: dark brown. Pterostigma color: mostly brown. Fore wing veins color: partially pigmented (r, RS, 2M and (RS+M)b dark; remaining pale).

*Head*. Antenna length/body length: antenna 0.96−0.98 × as long as body (head to apex of metasoma). Ocular–ocellar line/posterior ocellus diameter: 1.5–2.03. Interocellar distance/posterior ocellus diameter: 1.82–2.23. Antennal flagellomere 2 length/width: 2.36–3.5. Antennal flagellomere 14 length/width: 1.83−1.91. Length of flagellomere 2/length of flagellomere 14: 1.59–1.9. Tarsal claws: simple. Metafemur length/width: 3.3−4.2. Metatibia inner spur length/metabasitarsus length: 0.57–0.66.

*Mesosoma*. Anteromesoscutum: mostly with deep, dense punctures (separated by less than 2.0 × their maximum diameter). Mesoscutellar disc: with shallow punctures scattered all over. Number of pits in scutoscutellar sulcus: 9. Maximum height of mesoscutellum lunules/maximum height of lateral face of mesoscutellum: 0.3–0.45. Propodeum: with prominent median carina, including transverse carina extending to spiracle; as well as two partial lateral carinae on the posterior half of the propodeum (which seem to define a partial areola). Sculpture: anterior 0.3 strongly rugose (carinae mostly radiating from strong longitudinal median carina), smooth and shiny, costula present.

*Wings*. Length of fore wing veins r/2RS: 0.75−1.22. Length of fore wing veins 2RS/2M: 1.44–1.78. Length of fore wing veins 2M/(RS+M)b: 1.25-1.38. *Pterostigma* length/width: 2.52−3.0. Point of insertion of vein r in pterostigma: clearly beyond half length of pterostigma. Angle of vein r with fore wing anterior margin: clearly outwards, inclined towards fore wing apex. Shape of junction of veins r and 2RS in fore wing: distinctly angled.

*Metasoma*. Mediotergite 1 shape: parallel–sided anteriorly, narrowing at midlength, slightly widened posteriorly. Mediotergite 1 width at anterior margin/width at posterior margin: 0.77−0.88. Mediotergite 1 sculpture: smooth and shiny, except for widely scattered puncture at lateral margin and more so in the posterior half. Mediotergite 2 width at posterior margin/length: 2.26–2.94. Mediotergite 2 sculpture: mostly smooth. Outer margin of hypopygium: wide, semi-transparent. Ovipositor thickness: slightly tapering apically. Ovipositor sheaths length/metatibial length: 0.16–0.17, rarely 0.21.

**Male.** As female.

#### Molecular data.

GenBank Accession numbers: KM875666, KT308157 and KT308158.

#### Distribution.

India: Himachal Pradesh (Shimla), Kerala (Kasaragod), and Meghalaya (Barapani).

#### Biology/ecology.

**Figure 7.**
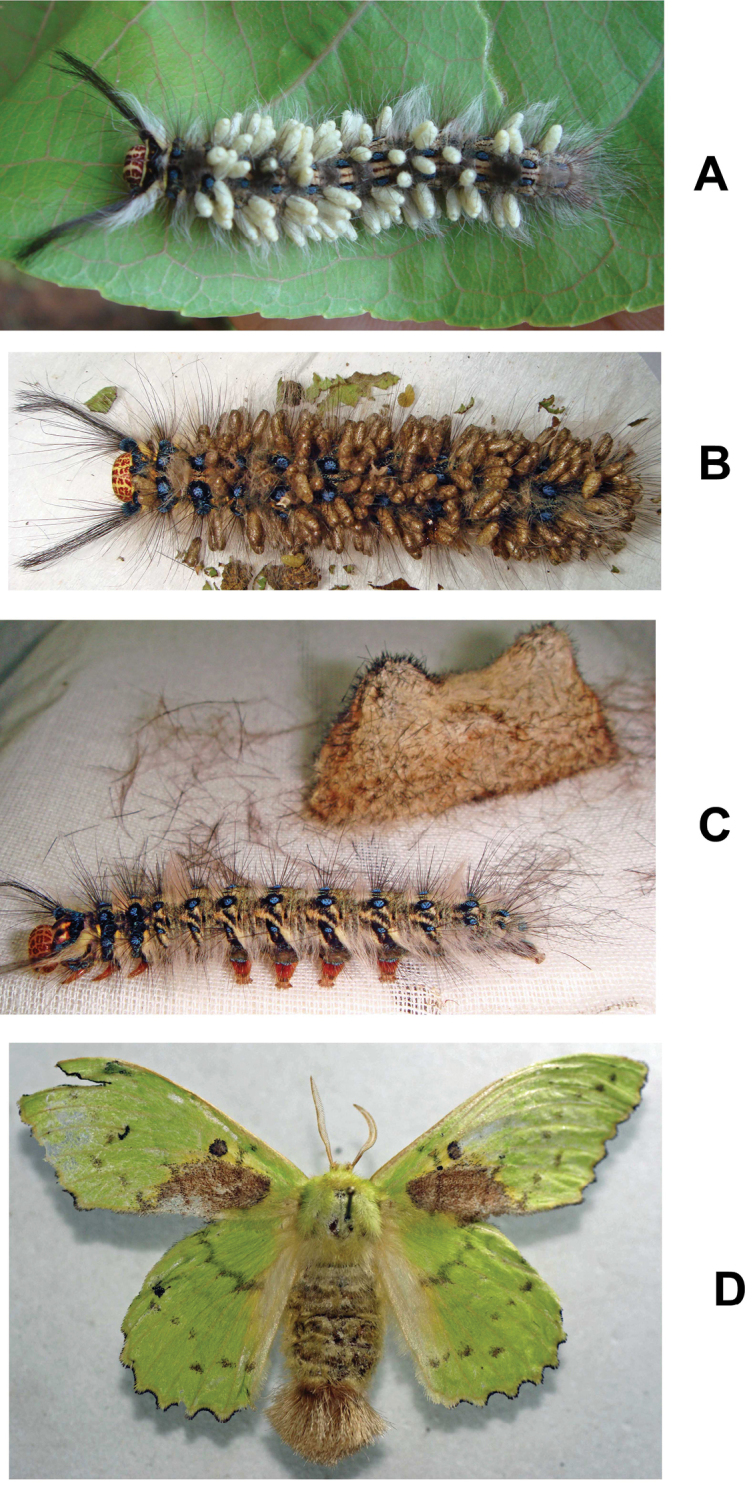
*Trabala vishnou* (Lefèbvre): **A** Parasitized caterpillar (Kasaragod) **B** Parasitized caterpillar (Shimla) **C** Unparasitized caterpillar and cocoon (Shimla) **D** Adult (Shimla).

Host (Fig. 7): *Trabala vishnou* (Lefèbvre) (Lasiocampidae) on *Ricinis communis* L. (in Meghalaya), *Rubus* sp. (in Shimla), and one indeterminate wild plant in southern India (in Kerala).

#### Etymology.

The name refers to the host species.

#### Comments.

General body coloration remains the same for all the populations, however minor variations were noticed: (i) south Indian population (from Kasaragod) has comparatively lesser ratio of ocular-ocellar line/posterior ocellus diameter: 1.50 *vs* 1.89−2.03 in both north Indian populations; (ii) ratio of mediotergite 1 width at anterior margin/width at posterior margin: >1 *vs* <1 in both northern populations; (iii) ratio of length of fore wing veins r/2RS: 1.22 *vs* 0.75−1.06 in both northern populations; (iv) T3 coloration remains the same as other tergites *vs* T3 yellowish brown in northern populations (more yellowish in Shimla population); (v) on an average ~70 white colored cocoons laid upright on a single host *vs* ~125 brown colored cocoons in both northern populations.

The reasons for the colour differences in the cocoons seen is not clear, but it might relate to different conditions (e.g. of humidity) pertaining at the time of their construction. The caterpillar with brown cocoons was collected from Shimla (northern India) which is humid in August while the caterpillar with white colored cocoons was collected in December from southern India (during the dry period).

**Figure 8.**
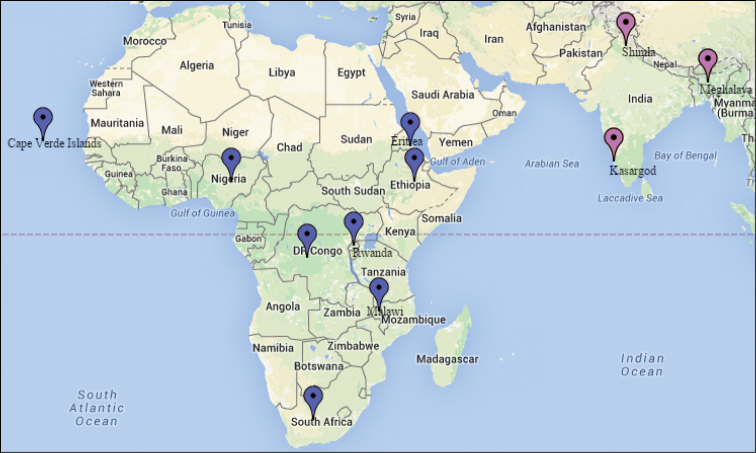
Map showing distribution of *Cotesia pistrinariae* (blue colored spots in Africa) and *Cotesia trabalae* (pink colored spots in India).

## Supplementary Material

XML Treatment for Cotesia trabalae
